# To Lumbar Puncture or Not to Lumbar Puncture: A Case of Lyme Neuroborreliosis

**DOI:** 10.7759/cureus.17970

**Published:** 2021-09-14

**Authors:** Carlos A Portales-Castillo, Mina Said

**Affiliations:** 1 Internal Medicine, Rochester Regional Health, Rochester, USA

**Keywords:** lyme disease and other tick borne pathogens, neurological lyme, tick-borne infections, bannwarth syndrome, neuroborreliosis

## Abstract

Lyme disease is a zoonotic infection increasing in prevalence across the United States. While the recognition of its classic clinical signs is sufficient to establish the diagnosis in the early stages, the diagnosis of Lyme neuroborreliosis (LNB) may be challenging and the diagnostic approaches may have to be tailored. We report a rare case of early disseminated LNB presenting with features of Banwarth syndrome in the form of painful radiculoneuritis, motor weakness, and facial palsy in a middle-aged female who presented to an Upstate New York Hospital during summer. Lyme antibody testing was found to be positive at a level of 11.70 by enzyme immunoassay and Western Blot was IgM positive with three out of three reactive borrelial proteins. Lumbar puncture was not performed per the patient’s preference. Otherwise, the laboratory workup along with MRI of the brain and cervical spine were grossly unremarkable. The patient was treated with a four-week course of oral doxycycline with resolution of all her symptoms. The diagnostic value of lumbar puncture in certain presentations of LNB remains controversial and is further discussed in this review.

## Introduction

Lyme disease is the most common vector-borne infection in the United States [1]. The geographical distribution of the disease has been expanding but still remains concentrated around the endemic areas, notably the Northeast, Mid-Atlantic, and upper Midwest regions [2]. The clinical manifestations of Lyme disease can vary by stage of infection. While the history of a tick bite in an endemic area between May and October along with the pathognomonic skin lesion of erythema migrans (EM) is sufficient to establish the diagnosis and warrant treatment, diagnosing multisystem involvement may prove to be challenging [3-6]. We present a rare case of early disseminated Lyme neuroborreliosis (LNB) with manifestations of facial palsy and painful radiculoneuritis as determined by clinical and serological criteria. Our case aimed to highlight the controversy on the diagnostic utility of cerebrospinal fluid (CSF) analysis in certain presentations of LNB.

## Case presentation

A 61-year-old Ukrainian woman with a past medical history of hypertension was admitted to the hospital in August for a 24-hour history of inability to completely close her left eye and a “funny feeling” on the left side of her face. Six weeks prior to presentation, she was seen at an urgent care center for erythema and swelling on the base of the index finger of her right hand after she thought she might have been bitten by a spider two days prior. On described examination, there was an erythematous raised circled rash on the dorsum of her right hand which was deemed to be cellulitis. She was prescribed a course of oral cephalexin 500 mg twice daily for seven days and was advised to use an over-the-counter nonsteroidal anti-inflammatory drug (NSAID) for the swelling. Three weeks after, she started to develop a progressively worsening posterior dull neck pain along with bilateral arm pain of similar characteristics radiating from her back to her shoulders which was not responsive to NSAIDs. The pain later became associated with progressive arm weakness, which continued to progress over the following three weeks up to the point where she could not comb her hair. Then, one day before presentation, left-sided facial weakness started and she decided to present to the hospital. On review of systems, she otherwise denied any fevers, chills, chest pain, shortness of breath, legs swelling, joint pains, vision changes, or other recent rashes. Her only home medication was amlodipine 10 mg daily. She had no known drug allergies. She denies any smoking, alcohol intake, or any other illicit drugs. She had no reported recent travel or hiking activities, but she enjoyed gardening in her big country house in Upstate New York. She did not have any pets or exposure to ticks that she was aware of. 

Physical examination on admission was notable for stable vital signs with a blood pressure of 125/80 mmHg, heart rate of 89 bpm, respiratory rate of 17/min, temperature of 36.8°C, and oxygen saturation of 97% on room air. Neurological examination revealed a complete flaccid left-sided facial palsy with no other cranial nerve involvement. The range of motion of both shoulders was limited on abduction to 45 degrees due to both pain and weakness. Examinations of the heart, lung, abdomen, skin, other joints, and the rest of the neurological signs were all unremarkable. 

On laboratory testing, complete blood count, serum electrolytes, thyroid-stimulating hormone (TSH), free T4, vitamin B12, urinalysis, and creatinine were within normal limits. Antinuclear antibody (ANA) test was negative, creatine kinase was 40 U/L (normal: 21-215 U/L), aldolase was 3.2 U/L (normal <7.7 U/L), sedimentation rate was 12 mm/h (normal: 0-30 mm/h), and C-reactive protein was <4.0 mg/L (normal: 0-10 mg/L). Imaging studies including brain MRI and head CT were unremarkable. MRI of the cervical spine showed mild cervical spondylosis with a normal-appearing spinal cord. On the basis of compatible neurological manifestations in an endemic region with a suspicious history of an arthropod bite weeks prior during the summer, Lyme antibody testing was sent and found to be positive at a level of 11.70 by enzyme immunoassay and Western Blot was IgM positive with three out of three reactive borrelial proteins. Diagnostic lumbar puncture (LP) for CSF analysis was recommended by the primary team for further workup. However, she politely declined as she wondered if the treatment decision would be altered by the results of an invasive procedure.

After discussion with the Neurology and Infectious Disease consultants, the diagnosis of early disseminated LNB manifesting as painful radiculoneuritis, motor weakness, and facial nerve palsy (so-called Bannwarth syndrome) was considered a strong possibility based on her clinical presentation and serologic criteria. She was subsequently discharged home on oral doxycycline 100 mg twice daily for four weeks along with oral prednisone 60 mg for five days and eye drops. After discharge, she was seen in the neurology office for follow-up at two weeks where she stated complete resolution of her facial weakness and pain along with marked improvement in her weakness which did not recur after successful course completion.

## Discussion

Lyme disease is a zoonotic infection mainly transmitted by the Ixodes scapularis, as well as other Ixodes ticks, and it represents the most common vector-borne infection in the United States [1]. The geographical distribution is typically confined to the Northeast, Mid-Atlantic, and upper Midwest regions of the United States and its incidence in these regions tends to follow a seasonal pattern with a predominant increase in the summer, particularly the months of June, July, and August [2].

The clinical manifestations are protean and depend on the stage of the diseases which are mainly categorized as early localized, early disseminated, and late disease. The early localized disease usually presents with erythema migrans, a classical round rash with occasional central clearing that typically appears between seven and 14 days after the tick bite and is present in up to 80% of cases, which at times can be accompanied by systemic symptoms suggestive of other viral infections [3,4]. It is estimated that only around 25% of patients presenting with EM recall a tick bite [3]. Early disseminated disease tends to present as either carditis, multiple EM lesions, generalized lymphadenopathy, or neurological features such as lymphocytic meningitis, radiculopathy, or unilateral or bilateral facial palsy. The late disease usually involves arthritis and persistent neurological presentations suggestive of encephalomyelitis. Data from the CDC suggest that regarding neurological manifestations, 9% of cases may present with facial palsy, 4% with radiculoneuropathy, and 2% with meningitis or encephalitis [1,2]. Classically described in Europe, the Bannwarth syndrome, a particular constellation of painful radiculoneuritis, various degrees of motor weakness, and occasional accompanying facial palsy, is thought to be less common in the United States and few case reports regarding this have been published [5].

LNB remains a challenging diagnosis and often warrants spinal fluid analysis, particularly in the context of acute meningitis. However, the ultimate decision to obtain a lumbar puncture in such patients with facial palsy and peripheral neurological symptoms remains controversial. The joint 2020 IDSA/AAN and ACR guidelines for the prevention, diagnosis, and treatment of Lyme disease differ significantly from the European Federation of Neurological Societies guidelines when it comes to the diagnosis of LNB. The former recommends an individualized approach for spinal fluid analysis, opting to favor serum antibody testing in the clinical scenario when a patient has a compatible presentation and risk factors, while the latter considers spinal fluid analysis as the cornerstone for diagnosing LNB in all of its forms [6,7].

Highly sensitive and specific tests of the CSF for LNB are still lacking. While a typical finding of lymphocytic pleocytosis with elevated protein is often seen and supports the diagnosis, these findings lack specificity as they are not uncommon in other nervous system infections. Other available testing options for the diagnosis of LNB include CSF antibodies to *Borrelia burgdorferi*, CSF:serum antibodies index, and in certain cases, CSF *B. burgdorferi* polymerase chain reaction (PCR). However, the sensitivities and specificities of these tests are variable and false-positive are common [6-11]. On the other hand, in some studies, up to >90% of patients with early LNB are seropositive by the traditional 2-tier serological test, which may obviate the need for a lumbar puncture (LP) in cases where acute meningitis is not suspected and the degree of suspicion for early disseminated LNB is high [8]. Furthermore, treatment with oral doxycycline for all early-disseminated neurological manifestations of LNB has been proven to be as effective as intravenous ceftriaxone making the treatment easy and the decision unlikely to be altered by the results of a fairly invasive test [9]. Nonetheless, CSF analysis does have a primordial role to exclude alternative diagnoses which can be the most concerning in certain circumstances.

Promising diagnostic assays with greater sensitivity and specificity such as the measurement of the CXCL13 chemokine biomarker in CSF for LNB are currently under development and when widely available, may reveal a higher value of obtaining an LP in the diagnosis and treatment of LNB [10]. However, the need for an LP in suspected cases of LNB remains a clinical decision that needs to be tailored to the specific clinical situation, favored when diagnostic uncertainty is present, and potentially spared when the clinical suspicion for LNB is high.

## Conclusions

Early disseminated Lyme neuroborreliosis is an uncommon diagnosis that requires a high degree of clinical suspicion and awareness. Major guidelines for diagnosing early disseminated Lyme neuroborreliosis differ significantly in the value and indications of lumbar puncture. Therefore, further studies on the clinical utility of CSF analysis in establishing the diagnosis, altering the treatment, and affecting the prognosis of Lyme neuroborreliosis with currently available and investigational tests are needed. 
